# Multidrug-Resistant Methicillin-Resistant Coagulase-Negative Staphylococci in Healthy Poultry Slaughtered for Human Consumption

**DOI:** 10.3390/antibiotics11030365

**Published:** 2022-03-09

**Authors:** Vanessa Silva, Manuela Caniça, Eugénia Ferreira, Madalena Vieira-Pinto, Cândido Saraiva, José Eduardo Pereira, José Luis Capelo, Gilberto Igrejas, Patrícia Poeta

**Affiliations:** 1 Microbiology and Antibiotic Resistance Team (MicroART), Department of Veterinary Sciences, University of Trás-os-Montes and Alto Douro (UTAD), 5000-801 Vila Real, Portugal; vanessasilva@utad.pt (V.S.); jeduardo@utad.pt (J.E.P.); 2 Department of Genetics and Biotechnology, University of Trás-os-Montes and Alto Douro, 5000-801 Vila Real, Portugal; gigrejas@utad.pt; 3 Functional Genomics and Proteomics Unit, University of Trás-os-Montes and Alto Douro (UTAD), 5000-801 Vila Real, Portugal; 4 LAQV-REQUIMTE, Department of Chemistry, NOVA School of Science and Technology, Universidade Nova de Lisboa, 2829-516 Caparica, Portugal; 5 National Reference Laboratory of Antibiotic Resistances and Healthcare Associated Infections (NRL-AMR/HAI), Department of Infectious Diseases, National Institute of Health Dr Ricardo Jorge, Av. Padre Cruz, 1649-016 Lisbon, Portugal; manuela.canica@insa.min-saude.pt (M.C.); eugenia.ferreira@insa.min-saude.pt (E.F.); 6 Centre for the Studies of Animal Science, Institute of Agrarian and Agri-Food Sciences and Technologies, Oporto University, 4051-401 Oporto, Portugal; 7 CECAV—Veterinary and Animal Research Centre, University of Trás-os-Montes and Alto Douro (UTAD), 5000-801 Vila Real, Portugal; mmvpinto@utad.pt (M.V.-P.); candido.ls95@gmail.com (C.S.); 8 Associate Laboratory for Animal and Veterinary Sciences (AL4AnimalS), 5000-801 Vila Real, Portugal; 9 BIOSCOPE Group, LAQV-REQUIMTE, Chemistry Department, Faculty of Science and Technology, NOVA University of Lisbon, 2825-466 Almada, Portugal; jlcm@fct.unl.pt; 10 Proteomass Scientific Society, 2825-466 Costa de Caparica, Portugal

**Keywords:** coagulase-negative *Staphylococcus*, CoNS, antimicrobial resistance, poultry, quails, broilers

## Abstract

Coagulase-negative staphylococci are commensals that are known to be prevalent in most environments, and they are also an important reservoir of antimicrobial-resistant genes. Staphylococcal infections in animal husbandry are a high economic burden. Thus, we aimed to determine the prevalence and species diversity of methicillin-resistant coagulase-negative staphylococci (MRCoNS) in poultry slaughtered for human consumption and to study the antimicrobial resistance of the isolates. Swab samples were recovered from 220 commercial chickens, homebred chickens and quails. Species identification was performed using MALDI-TOF. Antimicrobial susceptibility testing was performed by the disc diffusion method against 14 antimicrobials. The presence of antimicrobial-resistant genes was investigated by polymerase chain reaction. Totals of 11 (19.6%), 13 (20.3%), and 51 (51%) MRCoNS were isolated from commercial chickens, homebred chickens and quails, respectively. *S. lentus* was isolated from all homebred chickens, whereas 11 *S. lentus* and 2 *S. urealyticus* were isolated from commercial chickens. As for quails, the most prevalent MRCoNS were *S. urealyticus*. Almost all isolates had a multidrug-resistant profile and carried the *mecA* gene. Most isolates showed resistance to erythromycin, clindamycin, penicillin, tetracycline, ciprofloxacin and fusidic acid and harbored the *ermA*, *ermB*, *ermC*, *mphC* *tetK*, *tetL*, *tetM* and *tetO* genes. This study showed a frequent occurrence of multidrug resistance in MRCoNS isolated from healthy poultry in Portugal.

## 1. Introduction

Staphylococci colonize the skin and mucous membranes of humans and are considered commensals or opportunistic pathogens [1]. By 2018, 45 species and 24 subspecies of *Staphylococcus* had been described [2]. Staphylococci are divided into two groups, coagulase-positive (CoPS) and coagulase-negative staphylococci (CoNS), according to their ability to coagulate plasma. CoPS are pathogenic species which have the coagulase enzyme that converts plasma fibrinogen into fibrin [3]. CoNS lack this enzyme and were considered, until recently, to be minor pathogens or apathogenic [4]. CoNS possess fewer virulence factors that participate in the pathogenesis of infection when compared to CoPS, such as *S. aureus*, but, in the last few decades, CoNS have emerged as common causes of nosocomial infections [4]. Within the CoNS species, *S. epidermidis*, *S. haemolyticus* and *S. saprophyticus* are examples of the most significant types of CoNS in human infections [5]. As opportunistic pathogens, CoNS generally cause infection in colonized immunocompromised individuals, patients with catheters and prosthetic implants, dialysis and oncologic patients and neonates [6]. CoNS are responsible for a broad spectrum of infections, such as invasive endocarditis, bacteremia and bone infections [6,7]. In addition, increasing rates of antibiotic resistance have been detected in CoNS, in some cases even greater than for *S. aureus*, which limits the therapeutic options available [5]. Methicillin resistance in CoNS is usually due to the expression of the *mecA* gene, which encodes an alternative binding protein 2a (PBP2a) that has a low affinity for β-lactam antibiotics, although some studies have reported the presence the *mecC* gene, a homologue of *mecA* [8,9,10]. The *mec* genes are located on a mobile genetic element called the Staphylococcal Cassette Chromosome *mec* (SCC*mec*). SCC*mec* elements are more diverse in methicillin-resistant CoNS when compared to *S. aureus,* and many SCC*mec* elements could not be typed using multiplex PCR [10]. Tetracycline resistance is also frequently detected in different CoNS species [11].

CoNS also colonize and infect other mammals besides humans, with *S. chromogenes*, *S. simulans* and *S. xylosus* being the principal cause of infection [11]. CoNS are frequently responsible for arthritis, cow mastitis and, less often, systemic infections in animals [12]. The presence of CoNS has been reported in pets, livestock and wild animals [13,14,15]. It has been shown that food of animal origin can carry CoNS and other foodborne pathogens and, besides being able to cause infection, CoNS can also cause food poisoning [16]. Both CoPS and CoNS have been associated with avian pathologies such as arthritis, osteomyelitis, pododermatitis, septicemia and blepharitis [17,18]. Nevertheless, the presence of CoPS and CoNS has also been observed in healthy poultry and poultry meat, which may act as reservoirs and vehicles of zoonotic pathogens and antimicrobial resistance [16,19]. The spread of antimicrobial resistance among commensal CoNS in healthy poultry may represent a hazard for human and animal health [11]. Studies reporting the monitorization of antimicrobial-resistant pathogens in poultry and poultry meat have been published, but most studies focus only on *S. aureus* species [20,21,22,23,24]. The prevalence of antimicrobial-resistant pathogens in poultry, particularly staphylococci, may be due to their high consumption of antimicrobials. According to the ESVAC report, in Portugal the population-weighted mean consumption (expressed in milligrams per kilogram of estimated biomass) of antimicrobials was 175.8 mg/Kg in food-producing animals in 2020 [25]. In Portugal, the biomass-corrected consumption of third- and fourth-generation cephalosporins, quinolones, penicillin, macrolides and tetracyclines in food-producing animals was around 0.4, 7.3, 38.9, 20 and 60.4 mg/Kg [25]. Furthermore, all these antimicrobial classes were used in poultry production. Therefore, we aimed to investigate the presence of methicillin-resistant CoNS (MRCoNS) in healthy poultry for human consumption as well as the antimicrobial-resistant phenotypes and genotypes of the isolates.

## 2. Results

In this study, the presence of methicillin-resistant CoNS (MRCoNS) was detected in 71 (32.3%) of the 220 birds tested (Table 1). The co-carriage of two different species was identified in four animals, and 67 birds carried only one staphylococcal species. Co-carriage of MRCoNS species was identified only among quail samples, and the pattern of co-carriage was as follows: *Staphylococcus sciuri*/*S. urealyticus* (*n* = 2), *Staphylococcus lentus*/*S. urealyticus* and *Staphylococcus lentus*/*Staphylococcus haemolyticus*. A total of 75 MRCoNS were recovered and identified as *S. lentus* (*n* = 26), *S. urealyticus* (*n* = 21), *S. sciuri* (*n* = 15) and *S. haemolyticus* (*n* = 3). *S. haemolyticus* was exclusively isolated from quails. Chickens, both commercial and homebred, were mainly colonized by *S. lentus,* while *S. urealyticus* was the most frequently detected species in quails, followed by *S. lentus*. Quails were colonized significantly more frequently by MRCoNS than homebred chickens. Furthermore, the prevalence of *S. lentus* and *S. urealyticus* was significantly higher than that of *S. haemolyticus*. Results of the prevalence of each staphylococcal species are shown in Supplementary Figure S1.

**Figure 1:**
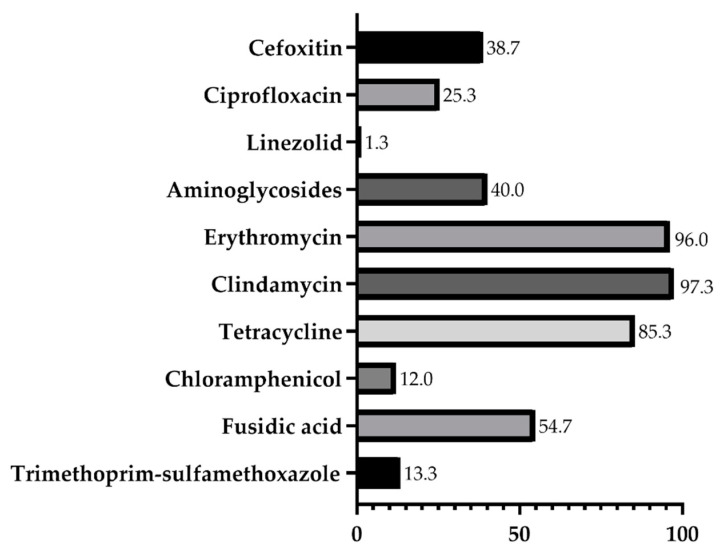
Percentage of resistance to each antibiotic by MRCoNS isolated from poultry.

Table 2 shows the antimicrobial-resistant phenotypes and genotypes of MRCoNS, while the detailed characterization of each isolate is summarized in Supplementary Table S1. The percentage of resistance to each antibiotic is shown in Figure 1. All isolates showed phenotypic and genotypic resistance to antibiotics, with 73 (97.3%) isolates displaying a multidrug-resistant profile since they showed resistance to at least three different classes of antimicrobials. The multidrug-resistance pattern was as follows: 15 (20%) isolates were resistant to 3 classes, 27 (26%) to 4 classes, 17 (22.7%) to 5 classes, 12 (16%) to 6 classes and 2 (2.7%) to 7 classes of antimicrobials. The non-multiresistant isolates were both *S. lentus* and were isolated from chickens. Both isolates showing resistance to seven antimicrobial classes were isolated from quails. The *mec*A gene was detected in all isolates, including those that were susceptible to cefoxitin. Totals of 11 *S. lentus*, 21 *S. urealyticus*, 14 *S. sciuri* and 3 *S. haemolyticus* were phenotypically resistant to penicillin, but the mechanism of penicillin resistance could not be identified. Resistance to aminoglycosides was detected in 40% of the isolates and was mediated by the *aph*(3′)-IIIa, *ant*(4′)-Ia and *str* genes in different combinations. All *S. lentus* and *S. urealyticus* were resistant to macrolides and lincosamides, while 14 *S. sciuri* and 2 *S. haemolyticus* showed resistance to this antimicrobial class. Macrolide-lincosamide resistant isolates harbored the *ermA*, *ermB*, *ermC* and *mphC* genes alone or in different combinations: *ermB* (*n* = 5); *ermC* (*n* = 11); *mphC* (*n* = 3); *ermC* and *mph*C (*n* = 27); *ermA*, *ermC* and *mphC* (*n* = 6); *ermB*, *ermC* and *mphC* (*n* = 10); *erm*B and *mphC* (*n* = 8); *ermA* and *ermC* (*n* = 1); *ermA*, *ermB*, *ermC* and *mphC* (*n* = 1); and *ermA*, *ermB* and *mphC* (*n* = 1). Tetracycline resistance, which was detected in all *S. urealyticus*, *S. sciuri* and *S. haemolyticus*, and in 25 (69.4%) *S. lentus*, was mediated by the tet*K*, tet*L*, tet*M* and/or *tetO* genes. The *tetL* gene was the most frequent, followed by the *tetK*. The *cat*_p194_ encoding resistance to chloramphenicol was detected in one *S. lentus* isolate. Resistance to trimethoprim-sulfamethoxazole was detected in 10 isolates. Some *S. lentus* isolates harbored a combination of *dfrK* and *dfrD* genes, while *S. sciuri* and *S. haemolyticus* carried only the *dfrK*. One *S. sciuri* exhibited resistance to linezolid, mediated by the *cfr* gene. None of the isolates showed resistance to vancomycin.

## 3. Discussion

MRCoNS in livestock was first reported in healthy chickens in Japan in 1996. Despite the increasing interest in CoNS in recent years, there is very limited information on their prevalence and resistance profiles in poultry production, and information is even more limited regarding MRCoNS. In our study, we investigated the presence of MRCoNS in healthy quails and commercial and homebred chickens. Among the 220 birds tested, 71 (32.3%) carried at least one CoNS, which is in accordance with the results obtained by Marek et al. [26]. CoNS colonized 47% and 20% of the quails and chickens, respectively. This carriage frequency was higher than the one obtained by Younis et al., who found a prevalence of CoNS in quails and chickens of 8.75% and 7.14%, respectively [27]. A study conducted with turkey samples found a frequency of CoNS of 15.6%, which is also lower than the one obtained in this study [28]. Other studies found a higher frequency of CoNS in poultry [18,29]. Nevertheless, it is important to point out that in our study all samples were only screened for the presence of MRCoNS, which may have contributed to a higher frequency of CoNS. Furthermore, some studies focused only on diseased animals that would most likely have been discarded in the slaughterhouse and would not have reached the final consumer. In our study, only four different species of CoNS were detected: *S. lentus* (*n* = 26), *S. urealyticus* (*n* = 21), *S. sciuri* (*n* = 15) and *S. haemolyticus* (*n* = 3). The predominant CoNS species found in our study included those commonly found in skin microbiota in chickens [29,30]. The occurrence of the staphylococci species among poultry samples appears to vary widely. *Pyzik* et al. detected a high number of CoNS species in diseased broiler chickens and turkeys, with *S. cohnii* being the most frequent followed by *S. saprophyticus* and *S. epidermidis* [29]. In accordance with our results, Saha et al. found a higher occurrence of *S. lentus* in poultry samples [30]. Boamah et al. reported a frequency of 42.97% *S. sciuri*, 35.94% *S. lentus*, 4.30% *S. xylosus*, 3.91%, *S. haemolyticus* 3.91%, 1.95% *S. saprophyticus* and 0.39% *S. cohnii* [31]. A study conducted in Brazil found that most CoNS from chickens were *S. gallinarum* followed by *S. simulans* [18]. In a report by El-Nagar et al., the majority of CoNS were *S. xylosus* [32]. Marek et al. found a higher occurrence of S. epidermidis in poultry in Poland [26]. Finally, *S.* *hominis* followed by *S.* *xylosus* and *S.* *lentus* were the most frequently detected species in quail eggs [33]. Yet, most studies have reported the presence of *S. sciuri*, *S. lentus* and *S.* *cohnii*. It has been shown that some species of CoNS, such as *S. sciuri*, *S. xylosus* or *S. cohnii,* are considered important poultry pathogens, particularly when associated with antimicrobial resistance [29]. Furthermore, most of these CoNS species are considered an issue of meat safety rather than the classical poultry pathogens [29].

The most common species found among poultry in this study was *S. lentus*. This species is considered an animal pathogen and has been detected among livestock, pets, wild animals and retail meats [13,16,34,35]. Nevertheless, *S. lentus* has also been responsible for a wide range of human infections and its clinical relevance seems to be increasing [36]. *S. urealyticus* was the second most common CoNS species found in poultry and it was mostly detected in quail samples. This CoNS species has been regarded as a commensal organism and is not usually involved in severe infections [37]. *S. urealyticus* strains of animal origin were shown to have multiple phenotypic resistances and carry several antimicrobial resistance genes [38]. All CoNS isolated in this study harbored the *mecA* gene, and the methicillin resistance of the isolates was confirmed. However, most *S. lentus* and *S. sciuri* isolates were phenotypically susceptible to cefoxitin. It has been shown that the staphylococcal species belonging to the *S.* *sciuri* group, which include *S. sciuri*, *S. fleurettii*, *S. lentus*, *S. stepanovicii* and *S. vitulinus*, carry a close homologue to the *mecA* gene, which does not confer resistance to β-lactam antibiotics [39]. Accordantly, almost all *S. urealyticus* had phenotypic resistance to cefoxitin. Multidrug resistance was exhibited in almost all isolates, which is in accordance with other studies conducted with poultry samples [27,28,29]. Although the European Union banned the use of antibiotics for growth promotion in livestock in 2006, and several other measures have been taken since then, it is estimated that over 60% of all antimicrobials produced are used in livestock comprising poultry [40]. Higher resistance levels were detected among quails, including two isolates resistant to seven antimicrobial classes, which may be explained by the fact that in Portugal the legislation for antibiotics administration in quails is not as well-regulated as that for other poultry, such as chickens; thus, antibiotics may be administrated indiscriminately to quails, leading to an increase in antimicrobial resistance [20]. Only one isolate, *S. sciuri*, was resistant to linezolid and carried the *cfr* gene. This gene was first detected in a bovine *S. sciuri* [41]. Although uncommon, resistance to linezolid mediated by the *cfr* gene is worrisome, since this gene confers cross-resistance to phenicols, lincosamides, oxazolidinones, pleuromutilins and streptogramin A antibiotics [42,43]. Studies reporting the *cfr* gene in poultry identified it in *S. lentus*, *S. urealyticus*, *S. arlettae*. *sciuri* and *S. simulans* [39,44,45]. Furthermore, a low frequency of this gene has been reported in CoNS from poultry [39]. Resistance to macrolides and lincosamides was detected in all isolates, except for one *S. sciuri* and one *S.* *haemolyticus*, and it was mediated by the *ermA*, *ermB*, *ermC* and *mphC* genes. Both *ermC* and *mphC* genes were carried by 56 isolates. Phosphotransferases are encoded by the *mphC* gene which confers resistance to erythromycin and other macrolides but not to lincosamides [46]. Nevertheless, the *erm* genes confer cross-resistance to macrolides, lincosamides and streptogramins B [46]. Although the *ermA* and *ermC* genes are the most frequent *erm* genes in staphylococci, the *ermA* gene was only detected in the *S. lentus* and *S. urealyticus* isolates, while *ermB* was identified in all MRCoNS species in this study. Other studies reported similar results for the frequency of *erm* genes in poultry [28,39]. A study by Syed et al. investigated the resistance of staphylococci in poultry intestines and reported a lower frequency of resistance to macrolides and lincosamides, but the *erm*C gene was also the most prevalent [47]. In the same study, resistance to tetracycline was detected in more than half of the isolates encoded by the *tetK* and *tetM* genes [47]. In our study, resistance to tetracycline was detected in 85.3% of the isolates, including all *S. sciutri*, *S. urealyticus* and *S. haemolyticus*, and in 25 out of 36 *S. lentus*, which was similar to the findings of other studies [28,31,48]. The high frequency of tetracycline resistance in poultry samples may be due to the fact that, according to the ECDC/EFSA/EMA report, tetracycline and penicillin were the most prescribed antibiotics for food-producing animals in 2017 [49]. Among the genes that confer resistance to tetracycline, *tet*L (*n* = 50) was the most prevalent, followed by *tetK* (*n* = 45), *tetO* (*n* = 16) and *tetM* (*n* = 9). Similar results were obtained by Lee et al. in a study that investigated the *tet* genes in poultry meat [16]. In contrast, in a study by Nemeghaire et al. *tet*M was the most common gene among *S. sciuri* from healthy chickens [39]. However, due to the lack of studies investigating the prevalence of resistant genes in CoNS from poultry, it is difficult to make a direct comparison. Fusidic acid was detected in 54.6% of the isolates but none of the resistance genes tested were found, which suggests the presence of other resistant genes. Indeed, in a study by Chen et al. none of the fusidic acid-resistant *S. urealyticus* possessed *fusB*, *fusC* or *fusD* genes; instead, *S. urealyticus* isolates carried the novel *fusF* gene, which seems to be an intrinsic factor in *S. urealyticus* and may not be conserved in another subspecies [50]. Resistance to vancomycin was not detected in this study, which was unsurprising since vancomycin-resistant staphylococci are rare and, as far as we know, in Portugal there is only one study reporting a vancomycin intermediate-resistant *S. aureus* isolated from a human infection [51].

In general, penicillin and tetracycline are extensively used for the treatment of staphylococcal infections in poultry [52]. In our study, we also found higher levels of resistance to those antimicrobial agents. The ingestion of poultry meat contaminated with staphylococci may lead to food poisoning. Furthermore, the handling or ingesting of staphylococci contaminated meat is a potential risk factor for colonization by methicillin-resistant staphylococci [53]. Our findings show that the frequency of multidrug-resistant staphylococci in poultry is alarming and may represent a public health problem.

## 4. Materials and Methods

### 4.1. Sample Collection and Bacterial Isolates

During the month of February 2020, a total of 220 samples were collected from poultry in a Portuguese slaughterhouse. Swab samples were collected from the cloaca and trachea of 100 quails, 50 commercial chickens and 70 homebred chickens. Batches of quails, homebred and commercial chickens arrived at the slaughterhouse 3 days a week and around 36,000 quails, 3500 homebred and 8000 commercial chickens were slaughtered each day. Four samples were recovered from each batch. The swabs were inserted into tubes containing brain heart infusion (BHI) broth with 6.5% of NaCl and incubated at 37 °C under aerobic conditions for 24 h. The inoculum was then seeded onto ORSAB agar plates supplemented with 2 mg/mL of oxacillin, incubated at 37 °C and examined after 24 h to 48 h. Up to three colonies per plate with different colors and morphology were recovered and further investigated. The staphylococci species identification was performed by matrix-assisted laser desorption/ionization time-of-flight coupled to time-of-flight mass spectrometry (MALDI-TOF MS) (Bruker Daltonics, Bremen, Germany) as described by Dubois et al. [54].

### 4.2. Phenotypic Antibiotic Resistance Testing

Antibiotic susceptibility profiles were determined for all of isolates by the Kirby–Bauer disc diffusion method on Mueller Hinton agar. The tested antibiotics included: cefoxitin (30 μg), chloramphenicol 132 (30 μg), ciprofloxacin (5 μg), clindamycin (2 μg), erythromycin (15 μg), fusidic acid (10 133 μg), gentamicin (10 μg), kanamycin (30 μg), linezolid (10 μg), mupirocin (200 μg), penicillin (1 U), tetracycline (30 μg), tobramycin (10 μg), and trimethoprim/sulfamethoxazole 135 (1.25/23.75 μg). The diameter of the inhibition zones was measured for each antibiotic disk and recorded in millimeters. The interpretation of results followed the recommendations given in the European Committee on Antimicrobial Susceptibility Testing (EUCAST) 2019 guidelines with the exception of kanamycin that followed the Clinical and Laboratory Standards Institute (CLSI) 2017 recommendations. The minimal inhibitory concentrations (MICs) of vancomycin were determined by a standard broth microdilution method in sterile 96-well microplates according to the EUCAST guidelines. Briefly, bacterial suspension was adjusted to 0.5 McFarland standards and then diluted 1:20. Then, 50 µL of Mueller–Hinton broth, 50 μL of the antibiotic dilutions, and 5 μL of the inoculum were mixed and incubated at 37 °C for 24 h. Isolates showing a vancomycin MIC ≤ 4 µg/mL were considered susceptible and those showing an MIC > 4 µg/mL were classified as resistant. The reference strain *S. aureus* ATCC 25923 was used for quality control.

### 4.3. DNA Extraction

DNA extraction was performed as previously described. Briefly, 2 staphylococci colonies were suspended in 45 μL of Milli-Q water and 5 μL of lysostaphin (1 mg/mL) was added. The samples were incubated at 37 °C for 10 min, after which 45 μL of Milli-Q water, 150 μL of Tris-HCl (0.1 M) and 5 μL of proteinase K (2 mg/mL) were added. After 10 min of incubation at 67 °C, the samples were boiled at 100 °C for 5 min. The DNA was stored at −20 °C until use. The spectrophotometric quantification of DNA was carried out through the NanoDrop 1000 (Thermo Fisher Scientific, Waltham, MA, USA) [55].

### 4.4. Antimicrobial-Resistant Genes

The presence of antimicrobial-resistant genes was investigated in each isolate according to the phenotypic resistance. The detection of the following antimicrobial-resistant genes was performed in a ProFlex^TM^ PCR system (Applied Biosystems, Waltham, MA, USA): beta-lactams (*blaZ*, *mecA* and *mecC*), linezolid (*cfr*), aminoglycosides (*aac*(6′)*-*aph**(2″), *aph*(3′)-IIIa, *ant*(4′)-Ia and *str*), macrolides and lincosamide (*ermA*, *ermB*, *ermC*, *ermT*, *msr(A/B*), mphC, lnu*A*, lnu*B*, *vgaA and vgaB*), tetracycline (*tetK*, tet*M*, *tetL* and *tetO*), chloramphenicol (*fexA*, *fexB*, *cat_pC194_*, *cat_pC221_* and *cat_pC223_*), fusidic acid (*fusB*, *fusC* and *fusD*) and trimethoprim/sulfamethoxazole (*dfrA*, *dfrG*, *dfrK* and *dfrD*). The protocol used for DNA amplification was as follows: a final volume of 50 µL contained 39.7 µL of ultra-pure water, 5 µL 10× complete buffer (Bioron, Römerberg, Germany), 1 µL 25 mM MgCl2, 1 µL deoxynucleotides triphosphate, 1 µL of each primer, 0.3 µL DFS Taq DNA polymerase (Bioron) and 1 µL DNA sample at 10 pg/µL. Primer sequences and PCR programs for the same are given in Table S2. The concentration and purity of the extracted DNA was measured using a spectrophotometer and Nano-DropTM software (Thermo ScientificTM, Waltham, MA, USA). Positive and negative controls used in all the experiments belonged to the strain collection of the University of Trás-os-Montes and Alto Douro.

### 4.5. Statistical Analysis

Pearson’s chi-square test was used compare the carriage of *S. sciuri*, *S. lentus*, *S. urealyticus* and *S. haemolyticus* between the quails, the homebred chickens and the commercial chickens. The analyses were carried out using IBM SPSS Statistics, Version 26.0 (IBM Corp., Armonk, NY, USA) and significance was set at *p* ≤ 0.05.

## 5. Conclusions

MRCoNS are common bacteria found in healthy poultry in Portugal. *S. urealyticus* seems to be more prevalent in quails, while broiler chickens are more often colonized by *S. lentus*, indicating a separate epidemiology. The high frequency of MRCoNS isolates in this study may be due to the fact that these bacteria are colonizers of the normal skin flora of animals. However, the multidrug resistance found in almost all isolates indicates that MRCoNS in poultry may be an important reservoir of antimicrobial-resistant genes. This is of great concern for public health, since most antimicrobial resistances detected were antimicrobials commonly used in human medicine. Some measures to overcome antimicrobial resistance in poultry in Portugal should be taken into consideration, such as the education of poultry producers, limiting the availability of antibiotics and the application of strict legislation concerning antimicrobial prescription.

## Figures and Tables

**Table 1:** Number of animals sampled, frequency and diversity of CoNS species detected among healthy poultry.

Animal	Number of Animals Sampled	Number of CoNS Carriers (%)	Isolates Recovered	*S. lentus*	*S. urealyticus*	*S. sciuri*	*S. haemolyticus*
Quails	100	47 (47)	51	15	19	14	3
Commercial chickens	50	13 (26)	13	11	2	-	-
Homebred chickens	70	11 (15.7)	11	10	-	1	-
Total	220	71 (32.3)	75	36	21	15	3

**Table 2:** Antimicrobial-resistant genes identified among the CoNS isolated from poultry.

Species	Number of Isolates	Antimicrobial Resistance	
		Phenotype	Genotype
*S. lentus*	36	PEN^11^, FOX^4^, CIP^11^, CN^2^, TOB^14^, KAN^9^, ERY^35^, CD^36^, TET^25^, C^4^, FD^12^, SXT^6^	*mecA^36^*, *ermA^8^*, *ermB^8^*, *ermC^28^*, *mphC^29^*, *aph(3′)-IIIa^9^*, *ant(4′)-Ia^12^*, *str^2^*, *tetL^19^*, *tetK^14^*, *tetO^1^*, *tetM^2^*, *cat_p194_^1^*, *dfrK^6^*, *dfrD^2^*
*S. urealyticus*	21	PEN^21^, FOX^18^, CIP^3^, CN^4^, TOB^6^, KAN^5^, ERY^21^, CD^21^, TET^21^, C^3^, FD^17^	*mecA^21^*, *ermA^1^*, *ermB^7^*, *ermC^19^*, *mphC^16^*, *aph(3′)-IIIa^5^*, *ant(4′)-Ia^2^*, *str^2^*, *tetL^17^*, *tetK^18^*, *tetO^13^*, *tetM^4^*
*S. sciuri*	15	PEN^14^, FOX^6^, LNZ^1^, CIP^3^, TOB^8^, KAN^4^, ERY^14^, CD^14^, TET^15^, C^2^, FD^10^, SXT^2^	*mecA^15^*, *cfr^1^*, *ermB^9^*, *ermC^7^*, *mphC^9^*, *aph(3′)-IIIa^3^*, *ant(4′)-Ia^7^*, *str^1^*, *tetL^11^*, *tetK^12^*, *tetO^2^*, *tetM^3^*, *dfrK^1^*
*S. haemolyticus*	3	PEN^3^, FOX^1^, CIP^2^, TOB^2^, KAN^1^, ERY^2^, CD^2^, TET^3^, FD^2^, SXT^2^	*mecA^3^*, *ermB^1^*, *ermC^2^*, *mphC^2^*, *aph(3′)-IIIa^2^*, *ant(4′)-Ia^1^*, *str^1^*, *tetL^3^*, *tetK^1^*, *dfrK^1^*

Abbreviations. C: chloramphenicol; CD: clindamycin; CIP: ciprofloxacin; ERY: erythromycin; FD, fusidic acid; FOX: cefoxitin; PEN: penicillin; SXT: trimethoprim-sulfamethoxazole; TET: tetracycline; CN: gentamicin; KAN: kanamycin; TOB: tobramycin; LNZ: linezolid. Note: the superscript number after each antibiotic and gene indicates the number of strains showing resistance to that antibiotic and harboring that gene, respectively.

## Supplementary Materials

The following supporting information can be downloaded at https://www.mdpi.com/article/10.3390/antibiotics11030365/s1: Table S1: Antimicrobial-resistant phenotype and genotype and SCC*mec* typing of CoNS isolated from poultry. Table S2: Primer pairs used for molecular typing and detection of antimicrobial resistance genes in MRSA strains. Figure S1: Prevalence of each staphylococci specie in poultry samples. References [56,57,58,59,60,61,62,63,64,65,66,67,68,69,70,71] are cited in the Supplementary Materials.
