# Supplementary material for: Multidrug-Resistant Methicillin-Resistant Coagulase-Negative Staphylococci in Healthy Poultry Slaughtered for Human Consumption
Source: Antibiotics (Basel). 2022 Mar 9;11(3):365. doi: 10.3390/antibiotics11030365 (PMC8944763; doi:10.3390/antibiotics11030365)
Supplement: Supplementary file 1 [file antibiotics-11-00365-s001.zip › antibiotics-1580876-supplementary.pdf]

Supplementary Materials

Multidrug-resistant Methicillin-Resistant Coagulase-Negative Staphylococci in Healthy Poultry for Human Consumption

Vanessa Silva, Manuela Caniça, Eugénia Ferreira, Madalena Vieira-Pinto, Cândido Saraiva, José Eduardo Pereira, José Luis Capelo, Gilberto Igrejas, Patrícia Poeta

Table S1. Antimicrobial resistance phenotype and genotype, and SCC*mec* typing of CoNS isolated from poultry.

Isolate	CoNS species	Origin	Antimicrobial resistance	
			Phenotype	Genotype
**VS3026**	*S. lentus*	Homebred chicken	CIP, TOB, KAN, ERY, CD, TET	*erm*B, *aph*(3′)-IIIa, *ant*(4′)-Ia, *tet*L
**VS3027**	*S. lentus*	Homebred chicken	CIP, TOB, ERY, CD, TET	*erm*C, *ant*(4′)-Ia, *tet*K
**VS3028**	*S. lentus*	Homebred chicken	CIP, TOB, KAN, ERY, CD, SXT	*erm*B, *aph*(3′)-IIIa, *ant*(4′)-Ia, *dfr*K
**VS3029**	*S. lentus*	Homebred chicken	CIP, TOB, KAN, ERY, CD	*erm*C, *mph*C, *aph*(3′)-IIIa, *ant*(4′)-Ia
**VS3030**	*S. lentus*	Homebred chicken	CIP, ERY, CD, C, SXT	*erm*A, *erm*C, *mph*C, *dfr*D, *dfr*K, cat_{p194}
**VS3031**	*S. lentus*	Homebred chicken	CIP, ERY, CD	*mph*C
**VS3032**	*S. lentus*	Homebred chicken	PEN, CIP, ERY, CD, TET	*erm*C, *tet*L
**VS3033**	*S. lentus*	Homebred chicken	PEN, CIP, ERY, CD, TET	*erm*C, *mph*C, *tet*K
**VS3034**	*S. lentus*	Homebred chicken	PEN, TOB, KAN, ERY, CD, TET, FD	*erm*B, *erm*C, *mph*C, *aph*(3′)-IIIa, *ant*(4′)-Ia, *tet*L, *tet*K
**VS3035**	*S. lentus*	Homebred chicken	PEN, CIP, ERY, CD	*erm*C, *mph*C
**VS3036**	*S. sciuri*	Homebred chicken	PEN, TOB, KAN, ERY, CD, TET	*erm*B, *aph*(3′)-IIIa, *ant*(4′)-Ia, *tet*L, *tet*K
**VS3037**	*S. lentus*	Commercial chicken	CIP, ERY, CD, TET	*mph*C, *tet*L

**VS3038**	*S. lentus*	Commercial chicken	CIP, ERY, CD, FD	*erm*A, *erm*C, *mph*C
**VS3039**	*S. lentus*	Commercial chicken	PEN, CIP, ERY, CD	*erm*C
**VS3040**	*S. lentus*	Commercial chicken	PEN, FOX, CIP, CN, TOB, KAN, ERY, CD, TET	*erm*A, *erm*C, *aph*(3′)-IIIa, *tet*L, *tet*M
**VS3041**	*S. lentus*	Commercial chicken	PEN, FOX+D19:D21, ERY, CD, FD	*erm*C, *mph*C
**VS3042**	*S. lentus*	Commercial chicken	PEN, CIP, ERY, CD	*erm*A, *erm*C, *mph*C,
**VS3043**	*S. lentus*	Commercial chicken	PEN, CIP, ERY, CD	*erm*A, *erm*B, *erm*C, *mph*C
**VS3044**	*S. lentus*	Commercial chicken	PEN, ERY, CD	*erm*C, *mph*C
**VS3045**	*S. lentus*	Commercial chicken	PEN, ERY, CD, TET	*erm*A, *erm*C, *mph*C, *tet*L, *tet*M,
**VS3046**	*S. lentus*	Commercial chicken	PEN, FOX, CIP, TOB, KAN, TET, C, SXT	*erm*A, *erm*C, *mph*C, *aph*(3′)-IIIa, *ant*(4′)-Ia, *tet*L, *dfr*D, *dfr*K, *fex*A
**VS3047**	*S. lentus*	Commercial chicken	PEN, ERY, CD, TET	*erm*C, *mph*C, *tet*L
**VS3048**	*S. cohnii* spp. *urealyticus*	Commercial chicken	PEN, FOX, ERY, CD, TET, FD	*erm*B, *mph*C
**VS3049**	*S. cohnii* spp. *urealyticus*	Commercial chicken	PEN, ERY, CD, TET	*erm*A, *erm*C, *mph*C, *tet*L, *tet*M, *tet*K
**VS3050**	*S. lentus*	Quail	PEN, TOB, ERY, CD, TET, FD	*erm*A, *erm*B, *mph*C, *aph*(3′)-IIIa, *ant*(4′)-Ia, *tet*L, *tet*K
**VS3051**	*S. lentus*	Quail	PEN, FOX, CIP, CN, TOB, KAN, ERY, CD, TET, FD	*erm*C, *mph*C, *str*, *tet*K, *tet*O
**VS3052**	*S. lentus*	Quail	PEN, CIP, ERY, CD, TET, FD, SXT	*erm*C, *mph*C, *tet*L, *dfr*K
**VS3053**	*S. lentus*	Quail	CIP, ERY, CD, TET, C, FD	*mec*A, *erm*C, *mph*C,
**VS3054**	*S. lentus*	Quail	PEN, CIP, ERY, CD, TET, FD	*erm*C, *mph*C, *tet*K
**VS3055**	*S. lentus*	Quail	PEN, CIP, ERY, CD, TET, FD	*erm*C, *mph*C, *tet*K, *tet*L
**VS3056**	*S. lentus*	Quail	PEN, CIP, ERY, CD, TET, FD	*erm*C, *mph*C, *tet*K
**VS3057**	*S. lentus*	Quail	PEN, CIP, ERY, CD, TET, C, FD, SXT	*erm*C, *mph*C, *tet*L, *dfr*K

**VS3058**	*S. lentus*	Quail	PEN, CIP, TOB, ERY, CD, TET	*erm*B, *mph*C, *ant*(4′)-Ia, *tet*L, *tet*K
**VS3059**	*S. lentus*	Quail	PEN, ERY, CD, TET, FD	*erm*C, *tet*L, *tet*K
**VS3060**	*S. lentus*	Quail	PEN, CIP, TOB, ERY, CD, TET	*erm*B, *mph*C, *ant*(4′)-Ia, *tet*L, *tet*K
**VS3061**	*S. lentus*	Quail	PEN, TOB, ERY, CD, TET	*erm*C, *mph*C, *ant*(4′)-Ia, *tet*L, *tet*K
**VS3062**	*S. lentus*	Quail	PEN, TOB, KAN, ERY, CD, TET	*erm*C, *mph*C, *aph*(3′)-IIIa, *ant*(4′)-Ia, *tet*K, *tet*L
**VS3063**	*S. lentus*	Quail	PEN, ERY, CD, TET	*erm*C, *mph*C, *tet*L, *tet*K
**VS3064**	*S. lentus*	Quail	PEN, CIP, TOB, KAN, ERY, CD, TET, SXT	*erm*B, *mph*C, *aph*(3′)-IIIa, *ant*(4′)-Ia, *str*, *tet*L, *dfr*K
**VS3065**	*S. sciuri*	Quail	PEN, TOB, ERY, CD, TET, FD	*erm*B, *ant*(4′)-Ia, *tet*L, *tet*K
**VS3066**	*S. sciuri*	Quail	PEN, TOB, KAN, ERY, CD, TET, FD	*erm*B, *aph*(3′)-IIIa, *ant*(4′)-Ia, *tet*L, *tet*K, *tet*O
**VS3067**	*S. sciuri*	Quail	PEN, FOX, CD, TET, FD	*tet*K
**VS3068**	*S. sciuri*	Quail	PEN, CIP, TOB, ERY, CD, TET, FD	*erm*C, *tet*L, *tet*K
**VS3069**	*S. sciuri*	Quail	PEN, CIP, TOB, ERY, CD, TET, FD	*erm*C, *ant*(4′)-Ia, *str*
**VS3070**	*S. sciuri*	Quail	PEN, TOB, KAN, ERY, CD, TET, FD	*erm*B, *mph*C, *ant*(4′)-Ia, *ant*(4′)-Ia, *tet*L, *tet*K
**VS3071**	*S. sciuri*	Quail	PEN, CIP, ERY, CD, TET	*erm*C, *mph*C, *tet*K
**VS3072**	*S. sciuri*	Quail	PEN, FOX, ERY, CD, TET, C, FD	*erm*B, *erm*C, *mph*C, *tet*L, *tet*K, *tet*M
**VS3073**	*S. sciuri*	Quail	PEN, TOB, ERY, CD, TET	*erm*B, *mph*C, *ant*(4′)-Ia, *tet*L, *tet*K
**VS3074**	*S. sciuri*	Quail	PEN, CIP, TOB, KAN, ERY, CD, TET, SXT	*erm*C, *mph*C, *aph*(3′)-IIIa, *ant*(4′)-Ia, *tet*K
**VS3075**	*S. sciuri*	Quail	ERY, CD, TET, SXT	*ermB*, *mph*C, *tet*L, *dfr*K
**VS3076**	*S. sciuri*	Quail	PEN, FOX, ERY, CD, TET, FD	*erm*B, *erm*C, *mph*C, *tet*L, *tet*K, *tet*O, *tet*M
**VS3077**	*S. sciuri*	Quail	PEN, FOX, LNZ, ERY, CD, TET, C, FD	*cfr*, *erm*B, *erm*C, *mph*C, *tet*L, *tet*K, *tet*M

**VS3078**	*S. sciuri*	Quail	PEN, FOX, CD, TET, FD	*mph*C, *tet*L
**VS3079**	*S. cohnii* spp. *urealyticus*	Quail	PEN, FOX, ERY, CD, TET	*erm*C, *mph*C, *tet*L, *tet*K,
**VS3080**	*S. cohnii* spp. *urealyticus*	Quail	PEN, FOX, CIP, CN, ERY, CD, TET, C, FD	*erm*B, *erm*C, *mph*C, *tet*K, *tet*O
**VS3081**	*S. cohnii* spp. *urealyticus*	Quail	PEN, FOX, CN, TOB, ERY, CD, TET, FD	*erm*B, *erm*C, *mph*C, *ant*(4′)-Ia, *str*, *tet*K, *tet*L, *tet*O
**VS3082**	*S. cohnii* spp. *urealyticus*	Quail	PEN, FOX, ERY, CD, TET, FD	*erm*C, *tet*L, *tet*K, *tet*O
**VS3083**	*S. cohnii* spp. *urealyticus*	Quail	PEN, TOB, KAN, ERY, CD, TET	*erm*C, *mph*C, *aph*(3′)-IIIa, *ant*(4′)-Ia, *tet*K, *tet*L
**VS3084**	*S. cohnii* spp. *urealyticus*	Quail	PEN, FOX, ERY, CD, TET, FD	*erm*B, *mph*C, *tet*L, *tet*K, *tet*O, *tet*M
**VS3085**	*S. cohnii* spp. *urealyticus*	Quail	PEN, FOX, ERY, CD, TET, FD	*erm*C, *mph*C, *tet*L, *tet*K
**VS3086**	*S. cohnii* spp. *urealyticus*	Quail	PEN, FOX, CIP, CN, TOB, KAN, ERY, CD, TET, FD	*erm*B, *erm*C, *mph*C, *aph*(3′)-IIIa
**VS3087**	*S. cohnii* spp. *urealyticus*	Quail	PEN, FOX, ERY, CD, TET, FD	*erm*B, *erm*C, *mph*C, *tet*L, *tet*K, *tet*O, *tet*M
**VS3088**	*S. cohnii* spp. *urealyticus*	Quail	PEN, FOX, ERY, CD, TET, FD	*erm*C, *mph*C, *tet*L, *tet*K, *tet*O
**VS3089**	*S. cohnii* spp. *urealyticus*	Quail	PEN, FOX, ERY, CD, TET, FD	*erm*B, *erm*C, *mph*C, *tet*L, *tet*K, *tet*O
**VS3090**	*S. cohnii* spp. *urealyticus*	Quail	PEN, FOX, CN, TOB, KAN, ERY, CD, TET, FD	*erm*C, *mph*C, *aph*(3′)-IIIa, *str*, *tet*L, *tet*K, *tet*O
**VS3091**	*S. cohnii* spp. *urealyticus*	Quail	PEN, FOX, ERY, CD, TET, FD	*erm*C, *mph*C, *tet*L, *tet*K, *tet*O
**VS3092**	*S. cohnii* spp. *urealyticus*	Quail	PEN, FOX, ERY, CD, TET	*erm*C, *mph*C
**VS3093**	*S. cohnii* spp. *urealyticus*	Quail	PEN, FOX, ERY, CD, TET, FD	*erm*C, *tet*L, *tet*K, *tet*O
**VS3094**	*S. cohnii* spp. *urealyticus*	Quail	PEN, FOX, TOB, KAN, ERY, CD, TET, C, FD	*erm*C, *aph*(3′)-IIIa, *tet*L, *tet*K
**VS3095**	*S. cohnii* spp. *urealyticus*	Quail	PEN, FOX, ERY, CD, TET, FD	*erm*C, *mph*C, *tet*L, *tet*K, *tet*O, *tet*M
**VS3096**	*S. cohnii* spp. *urealyticus*	Quail	PEN, FOX, TOB, KAN, ERY, CD, TET, C, FD	*erm*C, *aph*(3′)-IIIa, *tet*L, *tet*K, *tet*O
**VS3097**	*S. cohnii* spp. *urealyticus*	Quail	PEN, CIP, ERY, CD, TET, FD	*erm*C, *tet*L, *tet*K, *tet*O

**VS3098**	*S. haemolyticus*	Quail	PEN, CIP, TOB, TET, FD	*aph*(3′)-IIIa, *tet*L
**VS3099**	*S. haemolyticus*	Quail	PEN, CIP, TOB, KAN, ERY, CD, TET, SXT	*erm*C, *mph*C, *aph*(3′)-IIIa, *ant*(4′)-Ia, *str, tet*L, *dfr*K
**VS3100**	*S. haemolyticus*	Quail	PEN, FOX, ERY, CD, TET, FD	*erm*B, *erm*C, *mph*C, *tet*L, *tet*K

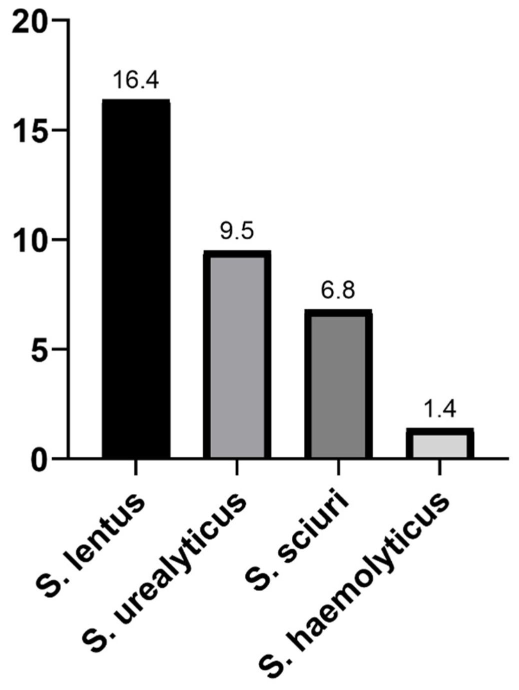

Figure S1. Prevalence of each staphylococci specie in poultry samples.

Table S2. Primer pairs used for molecular typing and detection of antimicrobial resistance genes in MRSA strains.

Gene (amplicon size)	Sequence (5′ › 3′)	Conditions	Reference
*mec*A (527 bp)	F: GGGATCATAGCGTCATTATTC<br>R: AACGATTGTGACACGATAGCC	94 ºC 5 min 1 cycle<br><br>94 ºC 30 sec<br>55 ºC 30 sec 30 cycles<br>72 ºC 1 min<br><br>72 ºC 10 min 1 cycle	[56]
*bla*Z (772 bp)	F: CAGTTCACATGCCAAAGAG<br>R: TACACTCTTGGCGGTTTC	94 ºC 3 min 1 cycle<br><br>94 ºC 1 min<br>50 ºC 1 min 30 cycles<br>72 ºC 2 min	[57]

		72 ºC 5 min 1 cycle	
*erm*A (645 bp)	F: TCTAAAAAGCATGTAAAAGAA<br>R: CTTCGATAGTTTATTAATATTAG	93 ºC 3 min 1 cycle	[58]
*erm*B (639 bp)	F: GAAAAGTACTCAACCAAATA<br>R: AGTAACGGTACTTAAATTGTTTA	93 ºC 1 min<br>52 ºC 1 min 35 cycles<br>72 ºC 1 min	[59]
*erm*C (642 bp)	F: TCAAAACATAATATAGATAAA<br>R: GCTAATATTGTTTAAATCGTCAAT	72 ºC 5 min 1 cycle	[58]
		94 ºC 3 min 1 cycle	
*erm*T (200 bp)	F: CCGCCATTGAAATAGATCCT<br>R: TTCTGTAGCTGTGCTTTCAAAAA	94 ºC 1 min<br>55 ºC 1 min 30 cycles<br>72 ºC 1 min	[60]
		72 ºC 5 min 1 cycle<br>95 ºC 3 min 1 cycle	
msr(A/B) (399 bp)	F: GCAAATGGTGTAGGTAAGACAACT<br>R: ATCATGTGATGTAAACAAAAT	93 ºC 30 sec<br>55 ºC 2 min 35 cycles<br>72 ºC 1,5 min	[61]
		72 ºC 5 min 1 cycle<br>94 ºC 3 min 1 cycle	
mph(C) (900 bp)	F: ATGACTCGACATAATGAAAT<br>R: CTACTCTTTCATACCTAACTC	94 ºC 1 min<br>45 ºC 1 min 30 cycles<br>72 ºC 1 min	[57]
		72 ºC 5 min 1 cycle<br>94 ºC 2 min 1 cycle	
lnu(A) (323 bp)	F: GGTGGCTGGGGGGTAGATGTATTAACTGG<br>R: GCTTCTTTTGAAATACATGG-TATTTTTCGATC	94 ºC 30 sec<br>57 ºC 30 sec 30 cycles<br>72 ºC 1 min	[62]
		72 ºC 10 min 1 cycle<br>94 ºC 5 min 1 cycle	
lnu(B) (944 bp)	F: CCTACCTATTGTTTGTGGAA<br>R: ATAACGTTACTCTCCTATTC	94 ºC 45 sec<br>54 ºC 45 sec 30 cycles<br>72 ºC 1 min	[63]
		72 ºC 5 min 1 cycle<br>94 ºC 3 min 1 cycle	
vga(A) (1264 bp)	F: AGTGGTGGTGAAGTAACACG<br>R: GGTTCAATACTCAATCGACTGAG	94 ºC 1 min<br>56 ºC 1 min 30 cycles<br>72 ºC 1 min	[64]

		72 ºC 5 min 1 cycle<br>94 ºC 1 min 1 cycle	
vga(B)<br>(576 pb)	F: TGACAATATGAGTGGTGGTG<br>R: GCGACCATGAAATTGCTCTC	94 ºC 1 min<br>55 ºC 2 min 30 cycles<br>72 ºC 2 min	[65]
		72 ºC 10 min 1 cycle<br>94 ºC 3 min 1 cycle	[66]
*tet*K<br>(697 bp)	F: TTAGGTGAAGGGTTAGGTCC<br>R: GCAAACTCATTCCAGAAGCA		
		94 ºC 1 min<br>54 ºC 1 min 30 cycles<br>72 ºC 1 min	[66]
*tet*M<br>(576 bp)	F: GTTAAATAGTGTTCTTGGAG<br>R: CTAAGATATGGCTCTAACAA		
		72 ºC 5 min 1 cycle<br>94 ºC 1 min 1 cycle	[66]
*tet*O<br>(615 bp)	F: GATGGCATACAGGCACAGAC<br>R: CAATATCACCAGAGCAGGCT		
*tet*L<br>(456 bp)	F: CATTTGGTCTTATTGGATCG<br>R: ATTACACTTCCGATTTCGG	94 ºC 1 min<br>50 ºC 1 min 30 cycles<br>72 ºC 1 min	[66]
		72 ºC 10 min 1 cycle<br>94 ºC 5 min 1 cycle	[67]
aac(6′)-Ie-*aph*(2′′)-Ia<br>(220 bp)	F: CCAAGAGCAATAAGGGCATA<br>R: CACTATCATAACCACTACCG	94 ºC 30 sec<br>60 ºC 45 sec 30 cycles<br>72 ºC 2 min	
		72 ºC 7 min 1 cycle<br>94 ºC 3 min 1 cycle	[67]
ant(4′)-Ia<br>(160 bp)	F: GCAAGGACCGACAACATTTC<br>R: TGGCACAGATGGTCATAACC	94 ºC 30 sec<br>60 ºC 45 sec 30 cycles<br>72 ºC 2 min	
		72 ºC 5 min 1 cycle<br>94 ºC 3 min 1 cycle	[57]
str<br>(646 bp)	F: TATTGCTCTCGAGGGTTC<br>R: CTTTCTATATCCATTCATCTC	94 ºC 1 min<br>50 ºC 1 min 30 cycles<br>72 ºC 1 min	
		72 ºC 5 min 1 cycle<br>94 ºC 3 min 1 cycle	[67]
aph(3′)-III<br>(292 bp)	F: GCCGATGTGGATTGCGAAAA<br>R: GCTTGATCCCCAGTAAGTCA	94 ºC 30 sec<br>60 ºC 45 sec 30 cycles<br>72 ºC 2 min	
		72 ºC 5 min 1 cycle<br>94 ºC 1 min 1 cycle	
*fex*A<br>(1272 bp)	F: GTACTTGTAGGTGCAATTACGGCTGA	94 ºC 1 min	[68]

	R: CGCATCTGAGTAGGACATAGCGTC	48 ºC 2 min 34 cycles	
		72 ºC 3 min	
		72 ºC 7 min 1 cycle	
		94 ºC 7min	
		min 1 cycle	
*fex*B	F: TTCCCACTATTGGTGAAAGGAT	94 ºC 1 min	
(816 bp)	R: GCAATTCCCTTTTATGGACGTT	55 ºC 1 min 30 cycles	[69]
		72 ºC 1 min	
		72 ºC 10 min 1 cycle	
cat$_{pC194}$	F: CGACTTTTAGTATAACCACAGA		
(570 bp)	R: GCCAGTCATTAGGCCTAT	94 ºC 3 min 1 cycle	[57]
cat$_{pC221}$	F: ATTTATGCAATTATGGAAGTTG	94 ºC 1 min	
(434 bp)	R: TGAAGCATGGTAACCATCAC	50 ºC 1 min 30 cycles	[57]
		72 ºC 1 min	
cat$_{pC223}$	F: GAATCAAATGCTAGTTTTAACTC	72 ºC 5 min 1 cycle	[57]
(283 bp)	R: ACATGGTAACCATCACATAC		
		94 ºC 3 min 1 cycle	
cfr	F: TGAAGTATAAAGCAGGTTGGGAGTCA	94 ºC 1 min	
(746 bp)	R: ACCATATAATTGACCACAAGCAGC	56 ºC 1 min 30 cycles	[70]
		72 ºC 1 min	
		72 ºC 5 min 1 cycle	
*fus*B	F: CTATAATGATATTAATGAGATTTTTGG	94 ºC 3 min 1 cycle	
(431 bp)	R: TTTTTACATATTGACCATCCGAATTGG	94 ºC 30 sec	
		57 ºC 30 sec 25 cycles	[70]
*fus*C	F: TTAAAGAAAAAGATATTGATATCTCGG	72 ºC 45 sec	
(332 bp)	R: TTTACAGAATCCTTTTACTTTATTTGG		
		72 ºC 7 min 1 cycle	
		94 ºC 7 min 1 cycle	
*fus*D	F: AATTCGGTCAACGATCCC	94 ºC 30 sec	
(456 bp)	R: GCCATCATTGCCAGTACG	57 ºC 30 sec 30 cycles	[71]
		72 ºC 30 sec	
		72 ºC 10 min 1 cycle	
*dfr*A	F: CCTTGGCACTTACCAAATG	94 ºC 3 min 1 cycle	
(374 bp)	R: CTGAAGATTCGACTTCCC	94 ºC 1 min	
		50 ºC 1 min 30 cycles	[57]
*dfr*D	F: TTCTTTAATTGTTGCGATGG	72 ºC 1 min	
(582 bp)	R: TTAACGAATTCTCTCATATATATG		
		72 ºC 5 min 1 cycle	
*dfr*G	F: TCGGAAGAGCCTTACCTGACAGAA	94 ºC 3 min 1 cycle	
(323 bp)	R: CCCTTTTTGGGCAAATACCTCATTCCA	94 ºC 1 min	[60]

		58 ºC 1 min 30 cycles<br>72 ºC 1 min<br><br>72 ºC 5 min 1 cycle
*dfr*K<br>(423 bp)	F: GAGAATCCCAGAGGATTGGG<br>R: CAAGAAGCTTTTCGCTCATAAA	94 ºC 3 min 1 cycle<br><br>94 ºC 1 min<br>56 ºC 1 min 30 cycles<br>72 ºC 1 min<br><br>72 ºC 5 min 1 cycle

[56] Zhang, K., Sparling, J., Chow, B.L., Elsayed, S., Hussain, Z., Church, D.L., Gregson, D.B., Louie, T., Conly, J.M., 2004, New quadriplex PCR assay for detection of methicillin and mupirocin resistance and simultaneous discrimination of Staphylococcus aureus from coagulase-negative staphylococci. J Clin Microbiol 42, 4947-4955.
[57] Schnellmann, C., Gerber, V., Rossano, A., Jaquier, V., Panchaud, Y., Doherr, M.G., Thomann, A., Straub, R., Perreten, V., 2006, Presence of new mecA and mph(C) variants conferring antibiotic resistance in Staphylococcus spp. isolated from the skin of horses before and after clinic admission. J Clin Microbiol 44, 4444-4454.
[58] Sutcliffe, J., Grebe, T., Tait-Kamradt, A., Wondrack, L., 1996, Detection of erythromycin-resistant determinants by PCR. Antimicrob Agents Chemother 40, 2562-2566.
[59] Shopsin, B., Mathema, B., Alcabes, P., Said-Salim, B., Lina, G., Matsuka, A., Martinez, J., Kreiswirth, B.N., 2003, Prevalence of agr specificity groups among Staphylococcus aureus strains colonizing children and their guardians. J Clin Microbiol 41, 456-459.
[60] Gomez-Sanz, E., Torres, C., Lozano, C., Fernandez-Perez, R., Aspiroz, C., Ruiz-Larrea, F., Zarazaga, M., 2010, Detection, molecular characterization, and clonal diversity of methicillin-resistant Staphylococcus aureus CC398 and CC97 in Spanish slaughter pigs of different age groups. Foodborne Pathog Dis 7, 1269-1277.
[61] Wondrack, L., Massa, M., Yang, B.V., Sutcliffe, J., 1996, Clinical strain of Staphylococcus aureus inactivates and causes efflux of macrolides. Antimicrob Agents Chemother 40, 992-998.
[62] Lina, G., Quaglia, A., Reverdy, M.E., Leclercq, R., Vandenesch, F., Etienne, J., 1999. Distribution of genes encoding resistance to macrolides, lincosamides, and streptogramins among staphylococci. Antimicrob. Agents Chemother. 43, 1062–1066.
[63] Bozdogan, B., Berrezouga, L., Kou, M.S., Yurek, D.A., Farley, K.A., Stockman, B.J., Leclercq, R., 1999. A new resistance gene, linB, conferring resistance to lincosamides by nucleotidylation in Enterococcus faecium HM1025. Antimicrob. Agents Chemother. 43, 925–929.
[64] Lozano C, Aspiroz C, Rezusta A, Gómez-Sanz E, Simon C, Gómez P, Ortega C, Revillo MJ, Zarazaga M, Torres C. Identification of novel vga(A)-carrying plasmids and a Tn5406-like transposon in meticillin-resistant Staphylococcus aureus and Staphylococcus epidermidis of human and animal origin. Int J Antimicrob Agents. 2012 Oct;40(4):306-12.
[65] Hammerum, A.M., Jensen, L.B., Aarestrup, F.M., 1998. Detection of the satA gene and transferability of virginiamycin resistance in Enterococcus faecium from food- animals. FEMS Microbiol. Lett. 168, 145–151.
[66] Aarestrup, F.M., Agers, L.Y., Ahrens, P., JC, J.L., Madsen, M., Jensen, L.B., 2000, Antimicrobial susceptibility and presence of resistance genes in staphylococci from poultry. Vet Microbiol 74, 353-364.
[67] Van de Klundert, J.A.M., Vliegenthart, J.S., 1993. PCR detection of genes coding for aminoglycoside-modifying enzymes, in: Persing, D.H., Smith, T.F., Tenover, F.C., White, T.J. (Eds.), Diagnostic Molecular Microbiology: Principles and Applications. American Society for Microbiology, Washington D.C., pp. 547–552.
[68] Kehrenberg, C., Schwarz, S., 2006. Distribution of Florfenicol Resistance Genes fexA and cfr among Chloramphenicol-Resistant Staphylococcus Isolates. Antimicrob. Agents Chemother. 50, 1156–1163.
[69] Liu, H., Wang, Y., Wu, C., Schwarz, S., Shen, Z., Jeon, B., Ding, S., Zhang, Q., Shen, J., 2012. A novel phenicol exporter gene, fexB, found in enterococci of animal origin. J. Antimicrob. Chemother. 67, 322–325.
[70] Mclaws, F., Chopra, I., O'Neill, A.J., 2008. High prevalence of resistance to fusidic acid in clinical isolates of Staphylococcus epidermidis. J. Antimicrob. Chemother. 61, 1040–1043.
[71] Chen, H.J., Hung, W.C., Tseng, S.P., Tsai, J.C., Hsueh, P.R., Teng, L.J., 2010. Fusidic acid resistance determinants in Staphylococcus aureus clinical isolates. Antimicrob. Agents Chemother. 54, 4985– 4991.
